# Transient exposure to calcium ionophore enables *in vitro* fertilization in sterile mouse models

**DOI:** 10.1038/srep33589

**Published:** 2016-09-15

**Authors:** Felipe A. Navarrete, Antonio Alvau, Hoi Chang Lee, Lonny R. Levin, Jochen Buck, Patricia Martin-De Leon, Celia M. Santi, Dario Krapf, Jesse Mager, Rafael A. Fissore, Ana M. Salicioni, Alberto Darszon, Pablo E. Visconti

**Affiliations:** 1 Department of Veterinary and Animal Science, Integrated Sciences Building, University of Massachusetts, Amherst MA, USA; 2 Department of Pharmacology, Weill Cornell Medical College, New York, NY, USA; 3 Department of Biological Sciences, University of Delaware, Newark, DE, USA; 4 Department of Obstetrics and Gynecology, Basic Sciences Division, Washington University School of Medicine. St. Louis, MO, USA; 5 Instituto de Biología Molecular y Celular de Rosario (CONICET-UNR), 2000 Rosario, Argentina; 6 Departamento de Genética del Desarrollo y Fisiología Molecular, IBT-UNAM, Cuernavaca, México

## Abstract

Mammalian sperm acquire fertilizing capacity in the female tract in a process called capacitation. At the molecular level, capacitation requires protein kinase A activation, changes in membrane potential and an increase in intracellular calcium. Inhibition of these pathways results in loss of fertilizing ability *in vivo* and *in vitro*. We demonstrated that transient incubation of mouse sperm with Ca^2+^ ionophore accelerated capacitation and rescued fertilizing capacity in sperm with inactivated PKA function. We now show that a pulse of Ca^2+^ ionophore induces fertilizing capacity in sperm from infertile *CatSper1* (Ca^2+^ channel), *Adcy10* (soluble adenylyl cyclase) and *Slo3* (K^+^ channel) KO mice. In contrast, sperm from infertile mice lacking the Ca^2+^ efflux pump PMACA4 were not rescued. These results indicate that a transient increase in intracellular Ca^2+^ can overcome genetic infertility in mice and suggest this approach may prove adaptable to rescue sperm function in certain cases of human male infertility.

In 1978, Steptoe and Edwards reported the birth of Louise Joy Brown, the first successful “Test-Tube” baby^1^. A major step toward this achievement occurred in the early 1950’s, when Chang^2^ and Austin^3^ demonstrated independently that sperm have to be in the female reproductive tract for a period of time before acquiring fertilizing capacity, a phenomenon now known as sperm capacitation. Capacitation includes all post-ejaculation biochemical and physiological changes that render mammalian sperm able to fertilize^4^. As part of capacitation, sperm acquire the ability to undergo acrosomal exocytosis^4^,^5^ and undergo changes in their motility pattern (i.e., hyperactivation). Molecularly, capacitation is associated with; (1) activation of a cAMP/protein kinase A pathway^6^; (2) loss of cholesterol^7^ and other lipid modifications^8^; (3) increase in intracellular pH (pH_i_)^9^; (4) hyperpolarization of the sperm plasma membrane potential^10^,^11^,^12^; (5) increase in intracellular Ca^2+^ concentration [Ca^2+^]_i_^13^; and (6) increase in protein tyrosine phosphorylation^14^,^15^. These pathways were first identified as playing a role in capacitation using compounds that either stimulate or block the respective signaling processes. More recently, the essential roles of cAMP, Ca^2+^ and plasma membrane hyperpolarization were confirmed using knock-out (KO) genetic approaches.

The role of cAMP in capacitation and fertilization was originally asserted using reagents such as cAMP agonists (dibutyryl cAMP, 8-BrcAMP) and antagonists of PKA-dependent pathways (e.g. H89, PKI, rpScAMP), as well as other conditions in which soluble adenylyl cyclase Adcy10 (aka sAC)^16^,^17^, the major source of cAMP in sperm, cannot be activated (e.g. HCO_3_^−^-free incubation media; addition of KH7, a specific sAC inhibitor)^18^. These roles of cAMP were confirmed using KO genetic mouse models lacking either the PKA sperm-specific catalytic splicing variant Cα2^19^, or sAC^18^; these mice are sterile and their sperm cannot fertilize *in vitro*. Our group has recently demonstrated that hyperpolarizing changes in membrane potential are necessary and sufficient to prepare the sperm for a physiological acrosome reaction^20^. Accordingly, sperm missing the sperm-specific K^+^ channel SLO3 cannot hyperpolarize and are infertile^21^. Finally, Ca^2+^ was shown to be essential for hyperactivation and the acrosome reaction both by removing it using Ca^2+^-free incubation media, either with or without chelating agents (i.e., EGTA)^22^, or by elevating it using Ca^2+^ ionophores such as A_23187_^23^. Consistent with these findings, male mice with the sperm-specific Ca^2+^ channel complex *CatSper* gene knocked out are infertile, and their sperm are unable to undergo hyperactivation^24^.

Recently, we found that addition of Ca^2+^ ionophore A_23187_ produced a fast increase in intracellular Ca^2+^ that was accompanied by complete loss of sperm motility^23^. However, if A_23187_ is removed after 10 min, intracellular Ca^2+^ levels dropped and sperm gained hyperactive motility^23^. In addition to inducing hyperactive motility, this short treatment with Ca^2+^ ionophore A_23187_ enhanced the sperm fertilizing capacity. Interestingly, the Ca^2+^ ionophore pulse supported capacitation in sperm incubated under non-capacitating conditions, and it induced hyperactivation and the capacity to fertilize *in vitro* even under conditions where cAMP-dependent pathways were blocked^23^. These results suggested that A_23187_ could overcome defects in the signaling pathways upstream of the increase in intracellular Ca^2+^ required for capacitation. Here, we tested this hypothesis using infertile genetic KO mouse models. Consistent with our hypothesis, a short A_23187_ pulse overcomes the infertile phenotypes of *CatSper*^24^, *sAC*^18^ and *SLO3* KO sperm^21^. Furthermore, our previous results suggested that after A_23187_ washout, sperm are required to reduce the intracellular Ca^2+^ concentrations to gain hyperactivation and fertilizing capacity^23^. Consistent with this hypothesis, sperm lacking the Ca^2+^ efflux pump PMCA4, which mediates Ca^2+^ extrusion^25^, were not rescued by treatment with ionophore, suggesting that this ATPase is required downstream to remove excess intracellular Ca^2+^.

## Results

### A_23187_ improves hyperactivation and fertilizing capacity of sperm from C57BL/6J mice

**Figure 1:**
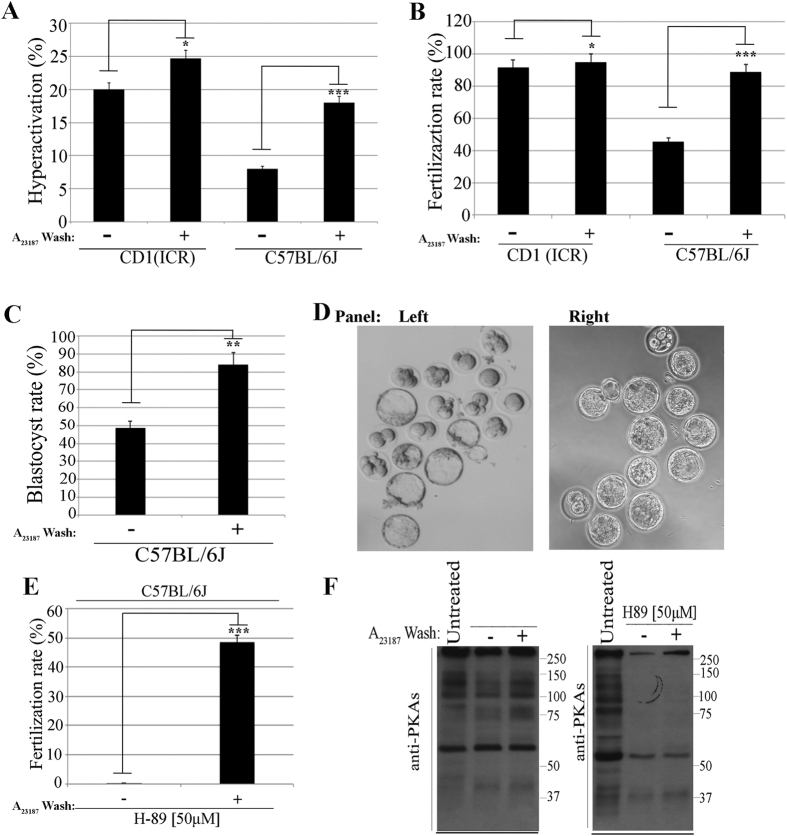
A_23187_ improves hyperactivation and fertilizing capacity of sperm from C57BL/6J genetic background. Sperm from CD1 (ICR) or C57BL/6J mice were treated with or without 20 μM A_23187_ for 10 min as described in Methods. After capacitation, sperm parameters were measured. In each of the panels, bars represent average ± SEM (*p < 0.05; **p < 0.01, ***p < 0.001) from independent experiments as indicated below. (**A**) Hyperactivation. The percentage of hyperactive motile sperm was obtained using CASAnova software (n = 4). (**B**) IVF. Fertilization rate was calculated considering the percentage of inseminated eggs achieving two-cell stage (n = 7). (**C**) Percentage of blastocyst formation. After 24 hours incubation, 2-cell embryos were transferred to KSOM media and incubated for additional 2.5 days to reach blastocyst stage. Notice that the percentage of blastocysts formation presented in the figure was obtained considering only the total 2-cell embryos and not the original number of oocytes. (**D**) Example of blastocysts formed using C57BL/6J sperm without (left panel) or with A_23187_ pre-treatment (right panel). (**E**) IVF conducted in the presence of H89 inhibitor. Sperm treated or not with A_23187_ for 10 min were incubated in the absence or in the presence of 50 μM H89. Fertilization rate was calculated as in B. (**F**) A_23187_ treatment overcomes the need for PKA activation in spermatozoa. Sperm treated or not with A_23187_ as described above were incubated in the absence or in the presence of 50 μM H89. Western blots were conducted as described in Methods (n = 3).

Sperm physiology and their ability to fertilize *in vitro* is highly dependent upon genetic background^26^. Over the years, C57BL/6J has been a common genetic background for studying KO genetic mouse models. Unfortunately, relative to sperm from mice of other genetic backgrounds, specifically CD1(ICR) mice, sperm from C57BL/6J exhibit significantly lower hyperactivation rates when capacitated^27^ (Fig. 1A, Supplementary Table I) and are less efficient for *in vitro* fertilization^26^ (Fig. 1B). When we compared the effect of a short pulse of Ca^2+^ ionophore on sperm from CD1 (ICR) with sperm from C57BL/6J mice, A_23187_ treatment elevated the percentage of hyperactive C57BL/6J sperm to similar levels as those obtained using CD1 (ICR) sperm (Fig. 1A). Moreover, this increase was followed by a significant increase in C57BL/6J sperm fertilization rate (Fig. 1B). Importantly, treating C57BL/6J sperm with a pulse of A_23187_ increased the percentage of 2-cell embryos competent to develop into blastocysts (Fig. 1C,D). Capacitation requires PKA activation^19^ and, as expected, in the presence of the PKA inhibitor H89, C57BL/6J sperm were unable to fertilize *in vitro* (Fig. 1E) and did not show the prototypical increase in phosphorylation of PKA substrates (Fig. 1F). Remarkably, as seen previously with CD1 (ICR) sperm^23^, incubating H89-treated C57BL/6J sperm for 10 min with A_23187_ was sufficient to induce fertilizing capacity (Fig. 1E), despite the fact that PKA remained inactive (Fig. 1F). Altogether, these data indicate that transient exposure to A_23187_ can improve IVF success for mouse strains with reduced fertility, in a PKA independent manner.

### A_23187_ treatment rescues hyperactivation and fertilizing capacity of *CatSper1* KO sperm

**Figure 2:**
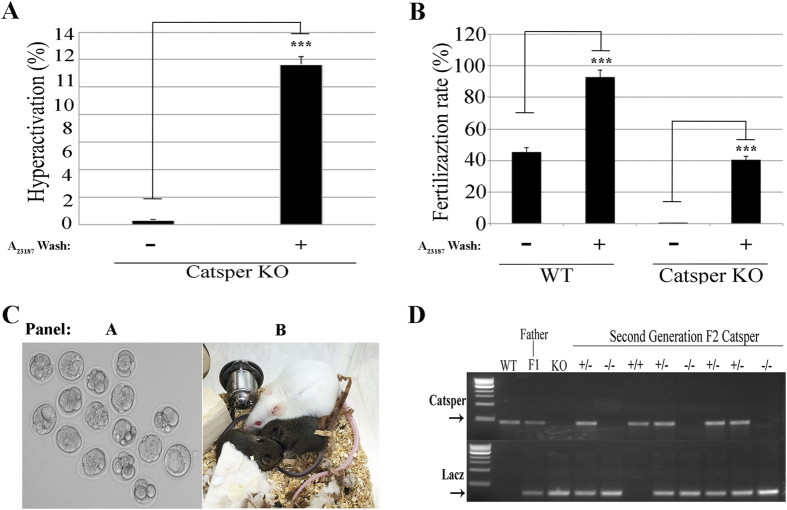
A_23187_ treatment induces hyperactivation and fertilizing capacity of *CatSper1* KO sperm. Mouse sperm from *CatSper* WT and KO were incubated in TYH medium in the presence or absence of A_23187_ as described above. In each of the panels, bars represent average ± SEM (*p < 0.05; **p < 0.01, ***p < 0.001) from 7 independent experiments. (**A**) Hyperactivation was measured in sperm from WT and *CatSper1*^*−/−*^ treated or not with a short (10 min) exposure to 20 μM A_23187_. After 1 hour and 20 minutes, sperm motility parameters were analyzed by CASA. (**B**) Approximately 1 × 10^6^ sperm cells from WT and KO *CatSper* were co-incubated with about 20–30 oocytes. Fertilization rate was scored 24 hour post-insemination as described above. (**C**) Two cell embryos from IVF were transferred to KSOM media and cultured for 2.5 more days until they reach late morula and early blastocyst (left panel). Then, blastocysts were non-surgically transferred to pseudo-pregnant females. 21 days later pups where born and reared to sexual maturity (right panel). (**D**) One heterozygous female and one heterozygous male were mated, and 8 F2 pups were born. The respective genotype from WT, F1, *CatSper*^−/−^ and F2 generations were analyzed by PCR.

In the absence of the CatSper channel complex, sperm fail to undergo hyperactivated motility and are unable to fertilize^24^. To test whether Ca^2+^ ionophore treatment can overcome the CatSper infertile phenotype, sperm from *CatSper1* KO mice were incubated in conditions that support capacitation in the absence or in the presence of 20 μM A_23187_. After 10 min, the sperm were washed twice by centrifugation in A_23187_-free media and the percentage of hyperactive sperm was measured using CASA. As expected, in the absence of A_23187,_ *CatSper* KO sperm did not undergo hyperactivation (Fig. 2A, Supplementary Table II and Supplementary Movie 1). However, once exposed to Ca^2+^ ionophore, a significant number of *CatSper* KO sperm exhibited hyperactivated motility (Fig. 2A, Supplementary Table II and Supplementary Movie 2). In addition, A_23187_-treated *CatSper* KO sperm were competent to fertilize metaphase II-arrested eggs *in vitro* (Fig. 2B). In two independent experiments, fertilized eggs were allowed to develop to late morula or blastocyst stage (Fig. 2C, left panel) and ten embryos in each case were non-surgically transferred to pseudopregnant WT female mice^28^,^29^,^30^. From these experiments, five CatSper (+/−) mouse pups were born from two different females (Fig. 2C, right panel). These heterozygous F1 mice were fertile; mating a male and female from this heterozygous population yielded a normal litter with 1 wild type, 4 heterozygous and 3 *CatSper* KO F2 progeny (Fig. 2D).

### A_23187_ treatment rescues hyperactivation and fertilizing capacity in sperm of *Adcy10 (aka sAC*) KO and *Slo3* KO but not in sperm from *Pmca4* KO mice

**Figure 3:**
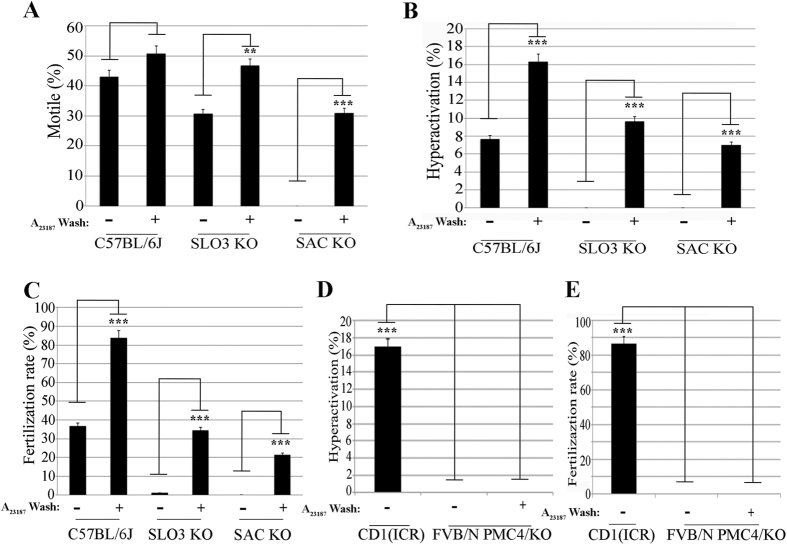
A_23187_ treatment induces fertilizing capacity in sperm from *Adcy10* and *Slo3* infertile KO genetic models but not in sperm from *Pmca4* KO. Sperm from 3 different KO genetic mice models with their respective WT were incubated in TYH standard in the presence or absence of A_23187_ as describe above. In each of the panels, bars represent average ± SEM (*p < 0.05; **p < 0.01, ***p < 0.001) from 7 WT (C57BL/6J), 3 *Slo3* KO and 4 *Adcy10* KO (aka sAC) independent experiments. (**A**) The percentage of motile sperm was measured by CASA system from WT (C57BL/6J), *Slo3* KO, and *Adcy10* KO (aka Sac) at time 1 hour and 20 min after A_23187_ treatment (10 min A_23187_ exposure). (**B**) Hyperactivation rate was measured at the same time by analysis of sperm motility parameters using CASAnova software. (**C**) Fertilization rate was scored 24 hour post-insemination as described above. (**D,E**) Analysis of sperm functional parameters in *Pmca4*^*−/−*^. Hyperactivation (**D**) and fertilization rate (**E**) were measured as above with sperm were pre-treated or not with A_23187_ for 10 min.

Capacitation requires up-regulation of cAMP concentrations^18^,^19^ and hyperpolarization of the sperm plasma membrane^21^. Under normal capacitation conditions, neither *sAC* KO nor *SLO3* KO sperm undergo hyperactivation (Fig. 3B), and while *SLO3* KO sperm are able to move (Supplementary Table III and Supplementary Movie 3), *sAC* KO sperm are almost immotile (Fig. 3A, Supplementary Table III and Supplementary Movie 5). Considering that transient exposure to A_23187_ can improve IVF success in a PKA independent manner (Fig. 1E and ref. 23), we tested whether these KO mouse models could be rescued by a Ca^2+^ ionophore pulse. When treated with A_23187_ for 10 min, a significant fraction of *sAC* KO sperm became motile and both *sAC* KO and *SLO3* KO sperm underwent hyperactivation (Fig. 3B and Supplementary Movies 4 and 6). Moreover, A_23187_ treatment induced *in vitro* fertilizing capacity in sperm from both KO models (Fig. 3C).

We previously showed that the increase in intracellular Ca^2+^ caused by A_23187_ has to be followed by a reduction in intracellular concentrations of this ion after removal of the ionophore^23^. In sperm, two molecules are thought to mediate Ca^2+^ extrusion, namely the Na^+^/Ca^2+^ exchanger and the more efficient, sperm-specific Ca^2+^ ATPase PMCA4^31^. Male *Pmca4* KO mice are infertile^32^; their sperm display poor motility and do not undergo hyperactivation (Fig. 3D,E). These data suggest this molecule is involved in regulation of normal Ca^2+^ homeostasis in sperm. We hypothesized that sperm lacking PMCA4 would have diminished capacity to efflux Ca^2+^ following ionophore treatment and be less susceptible to A_23187_ rescue. Treatment with A_23187_ rendered all *Pmca4*^*−/−*^ sperm motionless, and their motility was not recovered after ionophore removal (Fig. 3D). Consequently, neither their hyperactivated motility nor their fertilizing capacity was rescued (Fig. 3E).

## Discussion

Capacitation encompasses a series of sequential and concomitant biochemical changes required for sperm to gain full fertilization competency. Despite the relevance of capacitation, the molecular mechanisms intrinsic to this process are not well understood. A very early event in sperm capacitation is the activation of motility by a cAMP-dependent pathway^33^. The activation of cAMP synthesis occurs immediately after sperm are released from the epididymis and come into contact with high HCO_3_^−^ and Ca^2+^ present in the seminal fluid^34^,^35^. Plasma membrane transport of these ions regulates sperm cAMP metabolism through stimulation of Adcy10 (aka sAC)^18^, which elevates intracellular cAMP and activates PKA. Then, PKA phosphorylates target proteins and initiates several signaling pathways. These pathways include sperm plasma membrane hyperpolarization, increase in pH_i_, and increase in intracellular Ca^2+^ ions. Consistent with the influence of these events, KO mice models in which any of these pathways is interrupted are infertile.

Physiologically, sperm capacitation is associated with preparation for a physiological acrosome reaction and changes in their motility pattern collectively known as hyperactivation. Originally observed in hamster sperm moving in the oviduct, hyperactivated motility^36^ was later described in other mammalian species including humans^37^. Hyperactivation is associated with a strong, high-amplitude asymmetrical flagellar beating that appears to be essential for the sperm to loosen their attachment to the oviductal epithelium and to penetrate the zona pellucida^38^. Consistent with an essential role of hyperactivation for fertilization competency, low motility and/or defects in hyperactivation is one of the most common phenotypes observed in sperm from many different infertile knock-out models, including those used in the present work (i.e., *Catsper*^*−/−*^, *Adcy10*^*−/−*^, *Slo3*^*−/−*^ and *Pmca4*^*−/−*^)^18^,^21^,^24^,^32^.

Although very little is known about the molecular pathways regulating hyperactivation, Ca^2+^ ions have been shown to play roles in the initiation and maintenance of this type of movement^22^. Most of the information regarding the role of Ca^2+^ in hyperactivation has been obtained using loss-of-function approaches analyzing sperm motility in media devoid of Ca^2+^ ions. Gain-of-function experiments using Ca^2+^ ionophores (e.g. A_23187_, ionomycin) to increase [Ca^2+^]_i_ have yielded unexpected results because, instead of enhancing hyperactivation, these compounds stopped sperm movement^7^,^23^,^39^. Despite being motionless, ionophore-treated sperm are alive as they recover motility after the compound is quenched with lipophilic agents^39^ or removed by centrifugation^23^. The reversibility of the A_23187_ effect suggests that the sperm is able to return to physiological [Ca^2+^]_i_ after a drop in free ionophore concentration. In our previous work, we showed that a short incubation period with A_23187_, in addition to initiating hyperactivation, accelerated the acquisition of fertilizing capacity. Most importantly, our data indicated that 10 min incubation with A_23187_ induced fertilization competence even when activation of cAMP-dependent signaling pathways was blocked^23^.

Considering these results, we hypothesized that a temporary elevation of intracellular Ca^2+^ primes the sperm for hyperactivation and bypasses the need for other signaling pathways required to up-regulate Ca^2+^ influx in sperm. To test this hypothesis, in the present work, we selected four KO models affecting independent signaling pathways involved in sperm motility. Three of these signaling molecules are believed to act upstream of the increase in Ca^2+^ required for hyperactivation: CatSper, sAC and SLO3. Sperm from each of these mouse models were unable to undergo hyperactivation and are incapable of fertilizing metaphase II arrested eggs *in vitro*. In addition, *Pmca4* KO sperm were used, which would not allow intracellular Ca^2+^ lowering after saturating sperm cells with this ion. *Pmca4* KO mice are sterile because their sperm are deficient in both progressive and hyperactivated motility^25^,^40^. PMCA4 has been shown to be an essential source of Ca^2+^ clearance in sperm, and it is required to achieve a low resting [Ca^2+^]_i_^31^. Consistent with our hypotheses, a short incubation of sperm with A_23187_ induced hyperactivation of *CatSper*, *Adcy10* and *Slo3* KO but not of *Pmca4* KO sperm.

Male factors contribute to approximately half of all cases of infertility^41^. However, in over 75% of these cases it is unusual to have a clear diagnosis of the abnormalities found in semen parameters^42^,^43^. Currently, assisted reproductive technologies (ART) remain the main therapy available. Recent studies using KO mouse models, including those used in the present work, revealed that loss of function of a variety of genes results in infertility. Interestingly, several of these models display normal sperm counts, and their main deficiency is found in capacitation-associated processes such as impediments to undergo hyperactivation^24^, to undergo the acrosome reaction^21^, or to go through the utero-tubal junction *in vivo*^44^,^45^. We hypothesize that strategies designed to elevate [Ca^2+^]_i_ such as the use of A_23187_ pulse should overcome the need of upstream signaling pathways including but not limited to PKA activation. In addition, although IVF has been successfully employed in multiple species^5^, requirements of sperm for capacitation vary greatly among species and have been developed for each sperm type essentially by trial and error. In some species, such as the horse, effective methods for IVF have yet to be established despite decades of work^46^. Failure of equine IVF does not appear to be associated with oocyte characteristics^47^ but with the inability of horse sperm to hyperactivate and to penetrate the egg zona pellucida (**ZP**), two landmarks of capacitation. A better understanding of capacitation signaling processes have the potential to generate a “universal” IVF technology that can be used in endangered/exotic species for which ART is not currently available.

Improving IVF conditions would be of great value; however, at the clinical level, ICSI has replaced IVF when confronted with cases of infertility due to unknown male factor(s). ICSI is reliable and, from the patient’s point of view, more economical because of higher probability of success. Despite these advantages, ICSI bypasses certain aspects of normal fertilization and may bear effects that are not easily observed. Taking this into consideration, a method to improve IVF can be a desirable option in some male factor cases. It is worth noting that A_23187_ has already been used in the clinic for patients with repeated ICSI failure^48^ due to problems in egg activation. In these cases, fertilized eggs are transiently incubated with ionophore after ICSI, which exposes the zygote to high Ca^2+^. On the contrary, with the method described here, where sperm are transiently treated with A_23187_, the ionophore is washed out and does not come in contact with the embryo. More interestingly, using this methodology to overcome infertility problems related to motility and hyperactivation could be used to improve the success rate of intrauterine insemination, which is a significantly less invasive and less costly procedure than either IVF or ICSI.

## Methods

### Materials

Chemicals and other lab reagents were purchased as follows: Calcium Ionophore A_23187_ (C7522), Bovine serum albumin (BSA, fatty acid-free) (A0281), Tween-20 (P7949), fish skin gelatin (G7765), Pregnant mare serum gonadotropin (G4877) and human chorionic gonadotropin (CG5), were purchased from Sigma (St. Louis, MO). Non-Surgical Embryo Transfer (NSET) Device was acquired from Paratechs (Billerica, MA). N-[2-[[3-(4-bromophenyl)-2-propen-1-yl]amino]ethyl]5-isoquinolinesulfonamide, and dihydrochloride H-89 (130964-39-5) were purchased from Cayman chemical (Ann Arbor, Michigan). Embryo transfer light mineral oil (ES-005-C) and EmbryoMax® KSOM Medium (1X) w/1/2 Amino Acids (MR-106-D) were obtained from Millipore (Billerica, MA). Rabbit monoclonal anti-phosphoPKA substrates (anti-pPKAS) (clone100G7E), was purchased from Cell Signaling (Danvers, MA). Horseradish peroxidase-conjugated anti-rabbit IgGs was purchased from Jackson Immuno-Research Laboratories GE Life Sciences. 30% Acrylamide and β-Mercaptoethanol were obtained from Biorad.

### Animals

All procedures (including euthanasia, embryo transfer and genotyping) involving experimental animals were performed in accordance with Protocol #2013-0020 approved by the University of Massachusetts-Amherst Institutional Animal Care and Use Committee (IACUC). CD1 (ICR) mice were obtained from Charles River Laboratories (Wilmington, MA). Infertile KO mice genetic models (*CatSper* KO^24^, *Slo3* KO^21^, *Adcy10* KO^18^) and their corresponding wild type were on an C57BL/6J background; *Pmca4*^*−/−* ^32 mice and corresponding wild type were on an FVB/N background. These genetically modified mice models as well as their wild type siblings were either provided by authors of this manuscript (Dr. Levin and Dr. Buck for Adcy10−/−; Dr. Celia Santi for SLO3 KO; Dr. Patricia Martin-De Leon for PMCA4 KO) or donated (CatSper KO mice were donated by Dr. David Clapham). Three of these lines can also be obtained as cryopreserved embryos. The respective strain, stock number and respective website information are: Adcy10 KO: B6;129S5-*Adcy10*^*tm1Lex*^/Mmnc; Stock number: 011659-UNC (https://www.mmrrc.org/catalog/sds.php?mmrrc_id=11659). CatSper1 KO: B6.129S4-*Catsper1*^*tm1Clph*^/J; stock number: 018311 (https://www.jax.org/strain/018311). PMCA4 KO: *Atp2b4* nulls, MMRRC; Stock No: 36807-JAX (https://www.mmrrc.org/catalog/sds.php?mmrrc_id=36807). For CatSper embryo recipients, surrogate mothers were CD1 (ICR) females, 8–12 weeks of age. In experiments in which phosphorylation by PKA was investigated, C57BL/6J male mice were used. Vasectomized males were obtained from Charles River, and used to induce pseudopregnancy as previously described^49^.Non-surgical embryo transfer (NSET) was performed with an NSET device (ParaTechs, Lexington, KY)^29^,^30^.

### Media

Medium used for sperm capacitation and fertilization assays was Toyoda–Yokoyama–Hosi (standard TYH) medium^50^, containing 119.37 mM NaCl, 4.7 mM KCl, 1.71 mM CaCl_2_.2H_2_O, 1.2 mM KH_2_PO_4_, 1.2 mM MgSO_4_.7H_2_O, 25.1 mM NaHCO_3_^−^, 0.51 mM Na-pyruvate, 5.56 mM glucose, and 4 mg/mL bovine serum albumin (BSA), 10 μg/mL Gentamicin and phenol red 0.0006% at pH 7.4 equilibrated with 5% CO_2_. For capacitating conditions Ca^2+^ ionophore A_23187_ was used at a final concentration of 20 μM in TYH as previously described^23^.

### Mouse Sperm Preparation

Cauda spermatozoa were collected from each of the mouse strains described above. Each cauda epididymis was placed in 500 μL of TYH media. After 10 min. incubation at 37 °C (swim-out), epididymis tissue debris were removed, and the suspension adjusted to a final concentration of 1–2 10^7^ cells/ml and divided into two aliquots. Aliquots were supplemented with either 20 μM A_23187_ or equivalent quantities of DMSO (for controls) and further incubated at 37 °C. After 10 min. incubation, sperm were washed with 2 rounds of centrifugations (first one at 500 × g and the second one at 300 × g for 5 min each) in A_23187_-free TYH medium. Sperm were then re-suspended in A_23187_-free TYH and capacitated in CO_2_ incubator for an additional hour and 20 min. To evaluate sperm in conditions in which PKA is inactivated, H89 was used at a concentration of 50 μM for all incubation periods including those used for washing the ionophore A_23187_. After capacitation in each condition, sperm were used for the analysis of phosphorylated PKA substrates, hyperactivation and fertilizing capacity (see below).

### SDS-PAGE and Immunoblotting

After 1 hour and 20 min incubation in each condition, sperm proteins were extracted for Western blot analysis as previously described^22^. Protein extracts equivalent to 1 × 10^6^ sperm were loaded per line and subjected to SDS-PAGE an electro-transferred to PVDF membranes (Bio-Rad) at 250 mA for 90 min on ice. To analyze phosphorylated PKA substrates, anti-phosphoPKA substrate (anti-pPKAS) (1/10000) Western blots were used as described^22^.

### Hyperactive and Motility Parameters

Sperm suspensions (25 μl) were loaded into one pre-warmed chamber slide (depth, 100 μm) (Leja slide, Spectrum Technologies) and placed on a microscope stage at 37 °C. Sperm movements were examined using the CEROS computer-assisted semen analysis (CASA) system (Hamilton Thorne Research, Beverly, MA). The default settings include the following: frames acquired: 90; frame rate: 60 Hz; minimum cell size: 4 pixels; static head size: 0.13–2.43; static head intensity: 0.10–1.52; static head elongation: 5–100. Sperm with hyper activated motility, defined as motility with high amplitude thrashing patterns and short distance of travel, were sorted and analyzed using the CASAnova software^27^. At least 20 microscopy fields corresponding to a minimum of 200 sperm were analyzed in each experiment.

### Sperm Motility Video Recordings

Sperm suspensions (25 μl) were loaded into one pre-warmed chamber slide (depth, 100 μm) (Leja slide, Spectrum Technologies). Videos were recorded for 15 seconds using an Andor Zyla microscope camera (Belfast, Northern Ireland) mounted on Nikon TE300 inverted microscope (Chiyoda, Tokyo, Japan) fitted with 20 times objective lenses. Sample temperatures were maintained at 37 °C using a Warm Stage (Frank E. Fryer scientific instruments, Carpentersville, Illinois).

### Mouse eggs collection and IVF assays

Metaphase II-arrested mouse eggs were collected from 6–8 week-old super ovulated CD1 (ICR) female mice (Charles River Laboratories) as previously described^22^. Females were each injected with 5–10 IU equine chorionic gonadotropin and 5–10 IU human chorionic gonadotropin 48 h apart. The cumulus-oocyte complexes (COC’s) were placed into a well with 500 μl of media (TYH standard medium) previously equilibrated in an incubator with 5% CO_2_ at 37 °C. Fertilization wells containing 20–30 eggs were inseminated with sperm incubated as described above in medium supporting capacitation with or without A_23187_ treatment (final concentration of 1 × 10^6^ cells/ml). After 4 h of insemination, eggs were washed and put in fresh media. The eggs were evaluated 24 h post-insemination. To assess fertilization the three following criteria were considered: 1) the formation of the male and female pronuclei; 2) the emission of the second polar body; and 3) two-cells stages.

### Embryo Culture, Embryo transfer and Mice Genotyping

Twenty-four hours post-insemination, fertilized 2 cell embryos were transferred to drops containing KSOM media and further incubated between 3.5 and 4.1 days. At this stage, the percentage of blastocyst formation was evaluated. In some cases, 10 to 20 blastocysts were transferred to 2.5 days post coitum (dpc) pseudo-pregnant CD-1 recipient females using the non-surgical uterine embryo transfer device^28^. Pseudo-pregnant CD-1 recipient females were obtained by mating with vasectomized males (obtained from Charles River) one day after *in vitro* fertilization. Only females with a clear plug were chosen as embryo recipients; late morula and early stage blastocysts were chosen to be transferred. Routine genotyping was performed with total DNA from tail biopsy samples from weaning age pups as templates for PCR using genotyping primers for CatSper gene forward [5′-TAAGGACAGTGACCCCAAGG-3′] and reverse [5′-TAAGGACAGTGACCCCAAGG-3′] and for the reporter gene Lacz forward [5′TGATTAGCGCCGTGGCCTGATTCATTC-3′] and reverse [5′-AGCATCATCCTCTGCATGGTCAGGTC-3′] as described by the original authors^24^.

### Statistical analysis

Data from all studies are analyzed using SIGMA plot software (www.sigmaplot.com). Data are expressed as the means ± S.E.M. The difference between mean values of multiple groups was analyzed by one-way analysis of variance (ANOVA) followed by Tukey’s test. Statistical significances are indicated in the Figure legends.

## Additional Information

**How to cite this article**: Navarrete, F. A. *et al.* Transient exposure to calcium ionophore enables *in vitro* fertilization in sterile mouse models. *Sci. Rep.* **6**, 33589; doi: 10.1038/srep33589 (2016).

## Supplementary Material

Supplementary Information

Supplementary Movie 1

Supplementary Movie 2

Supplementary Movie 3

Supplementary Movie 4

Supplementary Movie 5

Supplementary Movie 6
