# Supplementary material for: Transient exposure to calcium ionophore enables in vitro fertilization in sterile mouse models
Source: Sci Rep. 2016 Sep 15;6:33589. doi: 10.1038/srep33589 (PMC5024339; doi:10.1038/srep33589)
Supplement: Supplementary Information [file srep33589-s1.pdf]

Transient exposure to calcium ionophore enables *in vitro* fertilization in sterile mouse models

Felipe A. Navarrete[1], Antonio Alvau[1], Hoi Chang Lee[1], Lonny R. Levin[2], Jochen Buck[2], Patricia Martin-De Leon[3], Celia M. Santi[4], Dario Krapf[5], Jesse Mager[1], Rafael Fissore[1], Ana M. Salicioni[1], Alberto Darszon[6,*] and Pablo E. Visconti[1,*].

[1]Department of Veterinary and Animal Science, Integrated Sciences Building, University of Massachusetts, Amherst MA, USA

[2]Department of Pharmacology, Weill Cornell Medical College, New York, NY, USA

[3]Department of Biological Sciences, University of Delaware, Newark, DE, USA

[4]Department of Obstetrics and Gynecology, Basic Sciences Division, Washington University School of Medicine. St. Louis, MO, USA.

[5]Instituto de Biología Molecular y Celular de Rosario (CONICET-UNR), 2000 Rosario, Argentina,

[6]Departamento de Genética del Desarrollo y Fisiología Molecular, IBT-UNAM, Cuernavaca, México

[*] To whom all correspondence should be sent: Pablo Visconti, Department of Veterinary and Animal Sciences, University of Massachusetts, Integrated Sciences Building 427W, 661 North Pleasant Street, Amherst, MA 01003-9301; Tel: (413) 545-5565; Fax: (413) 545-6326; email: pvisconti@vasci.umass.edu.

Alberto Darszon, Departamento de Genética del Desarrollo y Fisiología Molecular, IBT-UNAM, Cuernavaca, México; email: darszon@ibt.unam.mx

Running title: A_{23187} overcomes sperm infertility in vitro in gene KO model

Supplemental Films.

Video 1: Non- treated *CatSper* KO sperm.

Video 2: A_{23187} treated *CatSper* KO sperm.

Video 3: Non- treated *Slo3* KO sperm.

Video 4: A_{23187} treated *Slo3* KO sperm.

Video 5: Non- treated *Adcy10* KO sperm.

Video 6: A_{23187} treated *Adcy10* KO sperm.

Supplementary Tables.

In all supplementary tables, data were obtained as described in Methods using the CEROS computer-assisted semen analysis (CASA) system (Hamilton Thorne Research, Beverly, MA) as previously described[1]. The default settings include the following: frames acquired: 90; frame rate: 60 Hz; minimum cell size: 4 pixels; static head size: 0.13-2.43; static head intensity: 0.10-1.52; static head elongation: 5-100. Sperm with hyper activated motility, defined as motility with high amplitude thrashing patterns and short distance of travel, were sorted and analyzed using the CASAnova software[2]. At least 20 microscopy fields corresponding to a minimum of 200 sperm were analyzed in each experiment. Values are means ± S.E.M obtained with sperm from n number of individual mice as indicated in the respective table below. Parameter percentages are obtained considering only motile sperm. VAP, average path velocity in μm/sec; VSL, straight line velocity in μm/sec; VCL, curvilinear velocity in μm/sec; ALH, amplitude of lateral head displacement in μm; BCF, beat cross frequency in Hz.

Supplementary Table I. CASANova analyses of sperm motility from CD1 and C57BL/6J mice strains incubated or not for 10 minutes with 20 μM A_{23187} as described in Methods.

1-) CD-1 (ICR) sperm without A_{23187} treatment (n = 4)

Total Motility	69% ± 0.115					
	VAP	VSL	VCL	ALH	BCF	%
Weak	37.30 ± 2.8	12.45 ± 0.1	80.78 ± 6.7	7.470 ± 0.5	39.38 ± 1.4	24
Slow	95.40 ± 3.3	40.17 ± 4.4	173.8 ± 8.5	12.89 ± 0.4	29.77 ± 1.1	15
Intermediate	190.1 ± 8.7	123.2 ± 2.9	376.3 ± 10.8	20.19 ± 3.1	17.88 ± 3.9	3
Progressive	152.4 ± 7.5	115.1 ± 8.9	256.2 ± 3.2	15.50 ± 0.4	24.72 ± 0.9	38
Hyperactive	154.8 ± 4.8	50.37 ± 4.6	291.1 ± 3.2	19.18 ± 0.4	31.16 ± 0.5	20

2-) CD-1 (ICR) sperm with A_{23187} treatment (n = 4)

Total Motility	72% ± 1.2					
	VAP	VSL	VCL	ALH	BCF	
Weak	42.47 ± 4.1	15.48 ± 2.1	99.63 ± 10	9.66 ± 1.4	40.00 ± 1.5	22
Slow	93.92 ± 2.1	40.99 ± 5.7	179.8 ± 9.7	12.9 ± 0.6	28.46 ± 0.4	15
Intermediate	142.8 ± 9.4	93.55 ± 9.8	312.5 ± 15	20.25 ± 2.1	21.92 ± 5.6	4
Progressive	145.6 ± 10	117.6 ± 11	236.0 ± 14	12.31 ± 1.3	21.90 ± 2.1	35
Hyperactive	170.3 ± 10	63.94 ± 8.1	352.4 ± 29	21.25 ± 1.5	29.76 ± 1.9	24

3-) C57BL/6J sperm without A_{23187} treatment (n = 4)

Total Motility	53% ± 2.3					
Group	VAP	VSL	VCL	ALH	BCF	%
Weak	37.63 ± 0.2	13.14 ± 0.3	90.43 ± 0.8	8.085 ± 0.01	43.74 ± 0.8	41
Slow	87.97 ± 3.2	44.48 ± 0.4	170.5 ± 3.5	13.28 ± 0.2	27.28 ± 1.0	13
Intermediate	168.3 ± 7.2	122.5 ± 2.5	344.8 ± 4.7	16.37 ± 1.6	19.47 ± 6.1	2
Progressive	126.5 ± 8.3	349.7 ± 0.3	140.5 ± 38.8	12.78 ± 1.9	28.70 ± 5.8	37
Hyperactive	149.3 ± 3.4	53.76 ± 2.5	302.3 ± 9.2	20.73 ± 0.66	32.13 ± 0.2	8

4-) C57BL/6J sperm with A_{23187} treatment (n = 4)

Total Motility	67% ± 1.8					
Group	VAP	VSL	VCL	ALH	BCF	%
Weak	34.61 ± 0.7	13.32 ± 0.1	79.52 ± 6.1	6.57 ± 1.1	41.33 ± 1.1	28
Slow	93.34 ± 1.1	43.29 ± 0.3	183.3 ± 1.2	13.72 ± 0.1	30.66 ± 0.9	9
Intermediate	180.5 ± 0.8	114.1 ± 1.8	350.5 ± 5.4	21.16 ± 1.2	24.75 ± 2.2	3
Progressive	150.9 ± 5.1	123.6 ± 4.1	248.6 ± 2.2	15.42 ± 0.2	23.30 ± 0.1	42
Hyperactive	149.1 ± 2.4	56.04 ± 1.2	305.6 ± 7.3	21.24 ± 0.3	33.16 ± 0.1	18

Supplementary Table II. CASANova analyses of sperm motility from CatSper KO mice strains incubated or not for 10 minutes with 20 µM A23187 as described in Methods.

1-) C57BL/6J CatSper KO sperm without A_{23187} treatment (n = 7)

Total Motility	47% ± 3.9					
Group	VAP	VSL	VCL	ALH	BCF	%
Weak	50.72 ± 2.2	13.90 ± 0.6	117.2 ± 0.8	9.44 ± 9.4	40.30 ± 40	56.7
Slow	76.83 ± 2	40.09 ± 4.6	150.5 ± 16	10.90 ± 10	27.62 ± 27	34
Intermediate	163.0 ± 28	106.9 ± 13	334.7 ± 23	19.91 ± 19	21.23 ± 21	1
Progressive	180.4 ± 16	152.7 ± 14	257.4 ± 25	15.70 ± 15	18.76 ± 18	8
Hyperactive	209.2 ± 17	54.79 ± 7.8	386.4 ± 3.6	22.29 ± 22	29.27 ± 29	0.3

1-) C57BL/6J CatSper KO sperm with A_{23187} treatment (n = 7)

Total Motility	64% ± 4.5					
	VAP	VSL	VCL	ALH	BCF	%
Weak	39.26 ± 5.4	12.88 ± 1.3	92.53 ± 10	8.27 ± 0.9	41.88 ± 1.8	38
Slow	85.92 ± 3.1	41.25 ± 4.1	167.0 ± 13	12.06 ± 0.6	32.08 ± 2.2	26
Intermediate	228.0 ± 28	157.6 ± 28	371.9 ± 0.1	21.50 ± 5.2	21.93 ± 4.2	3
Progressive	157.9 ± 5	128.2 ± 2.5	244.1 ± 7.7	13.79 ± 0.7	24.61 ± 0.0	21
Hyperactive	123.0 ± 22	48.55 ± 0.6	308.7 ± 5.2	16.77 ± 2.1	37.06 ± 2.1	12

Supplementary Table III. CASANova analyses of sperm motility from wild type C57BL/6J, SLO3 KO and Adcy10 (aka sAC) KO mice incubated or not for 10 minutes with 20 µM A23187 as described in Methods. Note that data using sperm from C57BL/6J mice were done independently from the ones shown in Table I.

1-) C57BL/6J sperm without A_{23187} treatment (n = 7)

Total Motility	45% ± 2.2					
Group	VAP	VSL	VCL	ALH	BCF	%
Weak	38.78 ± 0.3	14.05± 0.9	89.67 ± 1.1	10.0 ± 0.1	43.74 ± 0.8	48
Slow	80.89 ± 2.2	45.67 ± 0.2	175.5 ± 2.5	14.45 ± 0.6	27.28 ± 1.0	14
Intermediate	176.8 ± 4.2	125.5 ± 3.4	345.8 ± 3.7	17.37 ± 1.7	19.47 ± 6.1	2
Progressive	116.4 ± 10	149.7 ± 0.5	135.5 ± 18.8	13.78 ± 2	28.70 ± 5.8	30
Hyperactive	150.3 ± 3.9	51.45 ± 3.5	310.3 ± 7.2	22.73 ± 0.89	32.13 ± 0.2	7

2-) C57BL/6J sperm with A_{23187} treatment (n = 7)

Total Motility	52% ± 1.7					
Group	VAP	VSL	VCL	ALH	BCF	%
Weak	35.61 ± 0.5	14.42 ± 0.1	84.40 ± 7.3	7.58 ± 1.6	42.43 ± 2.1	28
Slow	90.34 ± 1.5	44.29 ± 0.3	187.5 ± 1.5	13.88 ± 0.1	31.26 ± 0.7	9
Intermediate	185.5 ± 0.4	119.1 ± 1.2	356.2 ± 4.4	21.16 ± 1.2	23.35 ± 1.5	3
Progressive	155.9 ± 4.4	122.6 ± 2.1	147.7 ± 2.4	12.42 ± 0.6	25.30 ± 1.3	44
Hyperactive	148.1 ± 3.5	57.04 ± 2.3	300.2 ± 8.3	21.89 ± 0.2	33.89 ± 0.3	16

3-) C57BL/6J SLO3 KO sperm without A_{23187} treatment (n = 3)

Total Motility	31% ± 8.8					
Group	VAP	VSL	VCL	ALH	BCF	%
Weak	37.85 ± 2.7	13.40 ± 2.3	89.07 ± 8.6	7.51 ± 0.2	34.72 ± 5.7	52
Slow	70.10 ± 3.1	40.41 ± 6.0	146.0 ± 7.9	8.45 ± 2	24.94 ± 3.3	33
Intermediate	0.00 ± 0	0.00 ± 0	0.00 ± 0	0.00 ± 0	0.00 ± 0	0
Progressive	176.5 ± 20.8	150.0 ± 29	272.6 ± 5	14.9 ± 0.8	13.45 ± 13	15
Hyperactive	0.00 ± 0	0.00 ± 0	0.00 ± 0	0.00 ± 0	0.00 ± 0	0

4-) C57BL/6J SLO3 KO sperm with A_{23187} treatment (n = 3)

Total Motility	47% ± 7.2					
Group	VAP	VSL	VCL	ALH	BCF	%
Weak	37.63 ± 1.8	13.21 ± 0.5	80.94 ± 10	5.80 ± 1.4	41.43 ± 5.6	49
Slow	84.20 ± 3.4	42.27 ± 0.1	159.5 ± 7.7	11.18 ± 0.0	24.08 ± 0.3	30
Intermediate	163.7 ± 29	96.61 ± 26	321.9 ± 63	16.51 ± 3.4	12.70 ± 12	1
Progressive	157.1 ± 13	132.8 ± 20	256.3 ± 19	13.00 ± 0.0	19.27 ± 4.9	11
Hyperactive	143.9 ± 4	59.90 ± 5.5	312.5 ± 36	18.14 ± 2	29.86 ± 2	10

5-) C57BL/6J SAC1 KO sperm without A_{23187} treatment (n = 4)

Total Motility	2% ± 0.23					
Group	VAP	VSL	VCL	ALH	BCF	%
Weak	28.10 ± 0.3	8.88 ± 0.8	74.98 ± 6.9	4.46 ± 0.6	30.71 ± 7.5	52
Slow	81.36 ± 9.9	52.38 ± 3.4	137.1 ± 31	5.61 ± 1.1	6.40 ± 6.4	48
Intermediate	0.00 ± 0	0.00 ± 0	0.00 ± 0	0.00 ± 0	0.00 ± 0	0
Progressive	0.00 ± 0	0.00 ± 0	0.00 ± 0	0.00 ± 0	0.00 ± 0	0
Hyperactive	0.00 ± 0	0.00 ± 0	0.00 ± 0	0.00 ± 0	0.00 ± 0	0

6-) C57BL/6J SAC1 KO sperm with A_{23187} treatment (n = 4)

Total Motility	31% ± 4.1					
Group	VAP	VSL	VCL	ALH	BCF	%
Weak	36.18 ± 1.8	12.23 ± 0.2	104.9 ± 2.9	9.16 ± 0.2	45.29 ± 2.3	39
Slow	76.82 ± 2.3	42.34 ± 9.1	169.3 ± 14	13.03 ± 0.3	31.78 ± 1.2	32
Intermediate	221.4 ± 33	157.6 ± 5.9	471.2 ± 8.8	23.12 ± 0.0	17.07 ± 1.6	2
Progressive	173.6 ± 17	148.0 ± 12	253.5 ± 21	14.48 ± 0.2	16.93 ± 5.2	20
Hyperactive	152.5 ± 6.9	56.33 ± 1.2	399.6 ± 12	23.31 ± 1.1	40.20 ± 1.2	7

1 Navarrete, F. A. *et al.* Biphasic Role of Calcium in Mouse Sperm Capacitation Signaling Pathways. *J Cell Physiol*, doi:10.1002/jcp.24873 (2015).

2 Goodson, S. G., Zhang, Z., Tsuruta, J. K., Wang, W. & O'Brien, D. A. Classification of mouse sperm motility patterns using an automated multiclass support vector machines model. *Biol Reprod* **84**, 1207-1215, doi:10.1095/biolreprod.110.088989 (2011).
